# Inhibition of IL-17A by secukinumab shows no evidence of increased *Mycobacterium tuberculosis* infections

**DOI:** 10.1038/cti.2017.34

**Published:** 2017-08-25

**Authors:** Michael Kammüller, Tsen-Fang Tsai, Christopher EM Griffiths, Nidhi Kapoor, Pappachan E Kolattukudy, Dominique Brees, Salah-Dine Chibout, Jorge Safi Jr, Todd Fox

**Affiliations:** 1 Translational Medicine-Preclinical Safety, Novartis Institutes for Biomedical Research, Basel, Switzerland; 2 Department of Dermatology, National Taiwan University Hospital, Taipei, Taiwan; 3 Dermatology Centre, Salford Royal Hospital, University of Manchester, Manchester Academic Health Science Centre, Manchester, UK; 4 Burnett School of Biomedical Sciences, College of Medicine, University of Central Florida, Orlando, FL, USA; 5 Novartis Pharmaceuticals Corporation, East Hanover, NJ, USA; 6 Novartis Pharma AG, Basel, Switzerland

## Abstract

Secukinumab, a fully human monoclonal antibody that selectively neutralizes interleukin-17A (IL-17A), has been shown to have significant efficacy in the treatment of moderate to severe psoriasis, psoriatic arthritis and ankylosing spondylitis. Blocking critical mediators of immunity may carry a risk of increased opportunistic infections. Here we present clinical and *in vitro* findings examining the effect of secukinumab on *Mycobacterium tuberculosis* infection. We re-assessed the effect of secukinumab on the incidence of acute tuberculosis (TB) and reactivation of latent TB infection (LTBI) in pooled safety data from five randomized, double-blind, placebo-controlled, phase 3 clinical trials in subjects with moderate to severe plaque psoriasis. No cases of TB were observed after 1 year. Importantly, in subjects with a history of pulmonary TB (but negative for interferon-γ release and receiving no anti-TB medication) or positive for latent TB (screened by interferon-γ release assay and receiving anti-TB medication), no cases of active TB were reported. Moreover, an *in vitro* study examined the effect of the anti-tumor necrosis factor-α (TNFα) antibody adalimumab and secukinumab on dormant *M. tuberculosis* H37Rv in a novel human three-dimensional microgranuloma model. Auramine-O, Nile red staining and rifampicin resistance of *M. tuberculosis* were measured. *In vitro*, anti-TNFα treatment showed increased staining for Auramine-O, decreased Nile red staining and decreased rifampicin resistance, indicative of mycobacterial reactivation. In contrast, secukinumab treatment was comparable to control indicating a lack of effect on *M. tuberculosis* dormancy. To date, clinical and preclinical investigations with secukinumab found no evidence of increased *M. tuberculosis* infections.

Secukinumab, a fully human monoclonal antibody that selectively neutralizes interleukin-17A (IL-17A), has been shown to have significant efficacy in the treatment of moderate to severe psoriasis, psoriatic arthritis and ankylosing spondylitis, demonstrating a rapid onset of action and sustained responses with a favorable safety profile.^1, 2, 3, 4^

By blocking critical mediators of innate and adaptive immunity, biotherapeutics may carry a risk of increased opportunistic infections.^5, 6, 7, 8, 9, 10, 11, 12, 13^ It has been shown that IL-17A has a role in immune defense in mucocutaneous barrier tissues,^14, 15, 16^ in particular to extracellular fungi such as *Candida albicans.*^6, 7, 8^ In psoriasis patients, a 52-week treatment with secukinumab showed a dose-dependent but overall low incidence of transient, mild to moderate mucosal and cutaneous candidiasis,^17^ which is controllable by standard therapy and did not lead to any discontinuations in phase 2 and 3 clinical studies in patients with moderate to severe plaque psoriasis. To date, no cases of reactivation of latent tuberculosis (TB) infection (LTBI) were observed.^17^ However, reports that IL-17A-producing γδT cells and CD4^+^ T cells might have protective or pathologic roles during different phases of *Mycobacterium tuberculosis* infection^15, 16, 18, 19, 20, 21, 22^ emphasize the need to further explore the role of IL-17A in this context.

Vaccination studies with *Mycobacterium bovis*(bacille Calmette-Guérin) show increased IL-17A responses,^23, 24, 25^ however T-cell frequencies and cytokine expression profiles did not correlate with protection against TB after bacille Calmette-Guérin vaccination.^25^ *M. tuberculosis* infections have been associated with increased IL-17A levels in patients with acute TB,^26^ in *in vivo* mouse models,^27, 28, 29, 30, 31, 32^ as well as in *in vitro* human peripheral blood mononuclear cell (PBMC) cultures.^33, 34, 35^ Early granuloma formation may be dependent on IL-17A,^28^ but IL-17A-induced neutrophil recruitment may also increase pathological lesions and bacterial burden in chronic pulmonary infections.^36^ Unlike the equivocal role of IL-17A in host resistance to *M. tuberculosis* infections,^18, 19, 20, 21, 22^ the importance of tumor necrosis factor-α (TNFα) in immunity to this intracellular pathogen is well established, as documented clinically by the association of anti-TNFα therapies with reactivation of LTBIs, in psoriasis and rheumatoid arthritis indications.^9, 10, 11, 12, 13^ In a recent investigation we compared side by side the effects of anti-IL-17A, anti-IL-17F or TNFα-neutralizing surrogate antibodies in a murine *M. tuberculosis* H37Rv infection model, and confirmed the importance of TNFα in immunity to *M. tuberculosis* infection, in contrast to the anti-IL-17 pathway.^37^

To further explore any associations of secukinumab with reactivation of LTBIs, we have retrospectively re-evaluated pooled phase 3 clinical trials in subjects with moderate to severe plaque psoriasis, who had a history of pulmonary TB or tested positive for latent TB at screening, and received secukinumab for 1 year. In addition, to more directly study the effect of secukinumab on dormant mycobacteria in comparison with an anti-TNFα antibody treatment, we utilized a novel *in vitro* human *M. tuberculosis* H37Rv three-dimensional microgranuloma model.^38, 39, 40, 41, 42^

## Results

### Subjects with a medical history of TB or LTBI showed no reactivation of TB during secukinumab treatment

**Figure 1:**
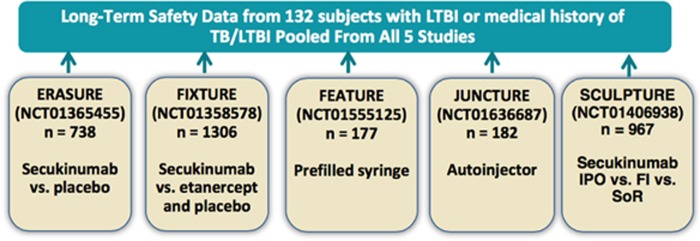
Safety data were pooled from five phase 3 studies of secukinumab in patients with moderate to severe plaque psoriasis. FI, fixed-interval; IPO, induction-period only; SoR, start of relapse.

**Figure 2:**
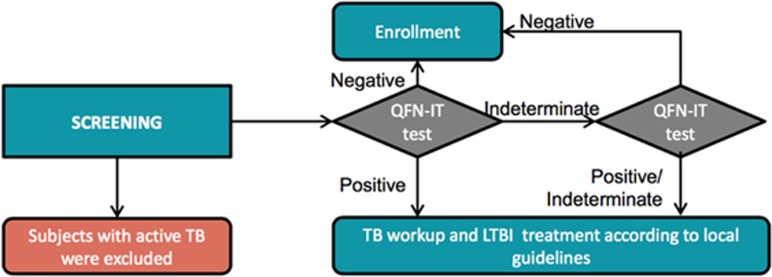
LTBI screening process in phase 3 trials. QFN-IT, Quantiferon-TB Gold in tube test.

Safety data were pooled across five secukinumab randomized, double-blind, placebo-controlled phase 3 clinical trials in 2044 subjects with moderate to severe plaque psoriasis to identify subjects with LTBI or previously treated TB and examine rates of reactivation (Figures 1 and 2). Subjects received secukinumab 300 or 150 mg subcutaneously at weeks 0, 1, 2 and 3, and then every 4 weeks starting from week 4 for 52 weeks, with an overall exposure of 2724.6 patient-years. Importantly, assessment of secukinumab safety (median duration 364 days) in 132 subjects with a history of treated pulmonary TB, revealed no reactivation of LTBI in 25 individuals, who tested negative by interferon-γ release assay (and receiving no anti-TB medication), and 107 subjects, who tested positive for LTBI, and hence received anti-TB medication (Table 1).

### Anti-TB therapy and secukinumab were taken concurrently with no serious adverse liver events

TB chemoprophylaxis (isoniazid and/or rifampin per local guidelines) is associated with elevated liver transaminases and gastrointestinal adverse events.^43, 44, 45^ Rates of liver enzyme abnormalities were low in secukinumab-treated subjects receiving treatment for LTBI diagnosed at screening (Table 2). In this analysis, 105 subjects received concomitant anti-TB therapy and secukinumab following LTBI diagnosis at screening or post randomization. No subjects discontinued secukinumab treatment while receiving chemoprophylaxis. Secukinumab was well tolerated in combination with anti-TB therapy in subjects who began chemoprophylaxis for LTBI before randomization.

### No reactivation of dormant *M. tuberculosis* in human *in vitro* microgranuloma model after anti-IL-17A treatment, in contrast to anti-TNFα treatment

**Figure 3:**
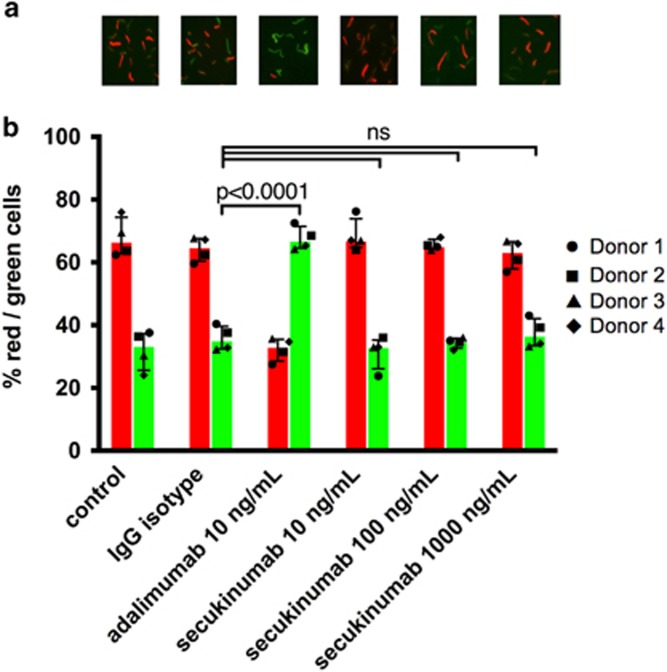
*M. tuberculosis* H37Rv inside hypoxic macrophages loses acid-fastness and accumulates lipid droplets, which is reversed by anti-TNFα (adalimumab), but not by anti-IL-17A (secukinumab) antibody treatment. PBMCs were infected with *M. tuberculosis* at multiplicity of infection 1:0.1. After granuloma formation (day 4), control IgG (10 ng ml^−1^), anti-TNFα (adalimumab) antibody (10 ng ml^−1^) or anti-IL-17A (secukinumab) antibody (10, 100 and 1000 ng ml^−1^) was added to the media. After 4 days of drug treatment *M. tuberculosis* cells from granuloma were obtained and stained with Auramine-O and Nile red staining. (**a**) Auramine-O and Nile red staining of *M. tuberculosis* cells recovered from hypoxic human macrophages. (**b**) The number of Auramine-O- and Nile red-positive *M. tuberculosis* cells were counted from multiple fields, and percentage of Auramine-O (green)- and Nile red (red)-positive *M. tuberculosis* cells are presented graphically. Figure represents percentage of red/green staining cells from four individual donors, with variances represented as median with interquartile ranges. Statistical analysis evaluated multiple comparisons with a two-way analysis of variance (GraphPad). Experiments were performed in an anonymized manner, and data are representative of two independent experiments with adalimumab and secukinumab and another anti-IL-17A antibody related to secukinumab, which also showed a lack of effect.

**Figure 4:**
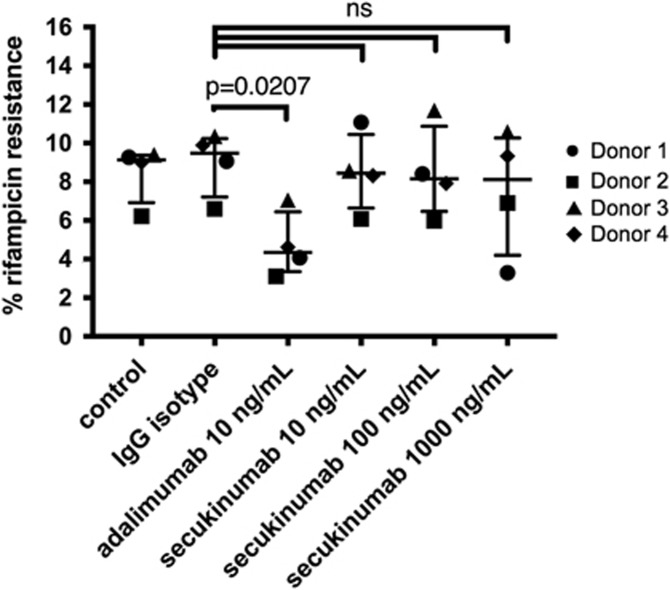
*M. tuberculosis* H37Rv inside hypoxic macrophages show rifampicin resistance, which is reversed by anti-TNFα (adalimumab), but not by anti-IL-17A (secukinumab) antibody treatment. PBMCs were infected with *M. tuberculosis* at multiplicity of infection 1:0.1. After granuloma formation (day 4), control IgG (10 ng ml^−1^), anti-TNFα (adalimumab) antibody (10 ng ml^−1^) or anti-IL-17A (secukinumab) antibody (10, 100 and 1000 ng ml^−1^) was added to the media. After 4 days of drug treatment rifampicin was added to the cultures and after 3 days *M. tuberculosis* cells from the granuloma were plated to determine the percentage of rifampicin resistance in four different donors. Figure represents percentage of rifampicin resistance from four individual donors, with variances represented as median with interquartile ranges. Statistical analysis evaluated multiple comparisons with a two-way analysis of variance (GraphPad). Experiments were performed in an anonymized manner, and data are representative of two independent experiments with adalimumab and secukinumab and another anti-IL-17A antibody related to secukinumab, which also showed a lack of effect.

As reported previously*, M. tuberculosis* exhibited the following characteristics when transitioning into dormancy from day 3 onwards: (a) loss of acid-fastness; (b) accumulation of lipid bodies; (c) development of rifampicin tolerance; as well as (d) gene expression changes.^38, 39, 40^ Human PBMCs from four healthy donors were infected *in vitro* with *M. tuberculosis* H37Rv at multiplicity of infection 0.1. Figures 3a and b show dual staining with Auramine-O and Nile Red, where acid-fast *M. tuberculosis* cells stain fluorescent green and lipid-body containing *M. tuberculosis* cells stain fluorescent red, respectively. The loss of acid-fastness and accumulation of lipid bodies indicate a dormant phenotype of this intracellular pathogen on day 8,^38, 39, 40, 41, 42^ and showed a ratio of about 65% red and 35% green staining for the isotype control group (Figure 3b). Moreover, rifampicin tolerance is a characteristic of dormant *M. tuberculosis.*^38, 39, 46^ Whereas <0.5% of *M. tuberculosis* cells in granuloma samples exhibit phenotypic rifampicin resistance on day 0, ~10% of the dormant mycobacteria show rifampicin resistance on day 8.^38, 39^ In our experimental conditions, dormant mycobacteria showed ~9% rifampicin resistance on day 8 (Figure 4).

Anti-TNFα antibody treatment can lead to reactivation of latent *M. tuberculosis* infections.^9, 10, 11, 12, 13^ Here we tested whether *M. tuberculosis* in cells of the *in vitro* microgranuloma can be reactivated by anti-TNFα adalimumab antibody treatment. Once the microgranuloma structures were formed *in vitro*, adalimumab was added to the media at 10 ng ml^−1^. Reactivation was assessed via changes in dormancy phenotypes, namely Auramine-O and Nile red staining patterns, and rifampicin resistance after 4 days of anti-TNFα antibody treatment. To determine the degree of dormancy of *M. tuberculosis* following drug treatment, microgranulomas were treated with 5 μg ml^−1^ rifampicin for 3 days followed by determination of colony-forming units (c.f.u.). Microgranulomas treated with a control IgG were used for comparison. The majority of *M. tuberculosis*-infected cells from microgranulomas treated with the control IgG remained positive for Nile red (median 65%), and fewer were positive for the Auramine-O stain (median 35% Figures 3a and b), pointing to a mostly dormant state of *M. tuberculosis*. However, a great majority of the *M. tuberculosis*-infected cells from microgranulomas treated with adalimumab were positive for the Auramine-O (median 67%) (Figures 3a and b). In addition, *M. tuberculosis* from microgranulomas treated with adalimumab also showed significantly less rifampicin resistance (median 4.3%) than mycobacteria from microgranulomas treated with the control IgG (median 9.5% Figure 4), indicating reactivation of *M. tuberculosis*. We did not find any significant difference in the structural morphology of the microgranulomas treated with a control IgG and anti-TNFα antibody (data not shown), which is in agreement with non-human primate studies demonstrating that TNFα neutralization results in disseminated disease in acute and latent *M. tuberculosis* infection while maintaining the granuloma structure.^47^

We added anti-IL-17A (secukinumab) antibody to the microgranulomas at 10, 100 and 1000 ng ml^−1^. After 4 days of anti-IL-17A antibody treatment microgranulomas were comparable with untreated or IgG control-treated microgranulomas, showing no effect on Auramine-O and Nile red staining patterns, and rifampicin resistance (Figures 3 and 4). This indicates that anti-IL-17A (secukinumab) antibody treatment *in vitro* did not reverse *M. tuberculosis* dormancy.

## Discussion

This observational study further supports that, to date, secukinumab treatment shows no evidence of an increase in *M. tuberculosis* infections based on clinical and *in vitro* findings. A retrospective re-evaluation of 1-year human phase 3 clinical trials with secukinumab revealed no evidence of increased incidence of acute TB and reactivation of LTBI. Importantly, secukinumab was well tolerated in combination with anti-TB therapy in subjects who began chemoprophylaxis for LTBI before randomization. Specifically, we did not find an increased incidence of elevated liver enzymes in isoniazid-treated patients^44, 45^ during secukinumab treatment. Whereas, elevated *M. tuberculosis* infection rates have been reported in association with anti-TNFα therapies in subjects with psoriasis and rheumatoid conditions,^9, 10, 11, 12, 13^ the low risk for TB reactivation upon secukinumab treatment in subjects with a medical history of TB or LTBI is further supported experimentally by the *in vitro* findings in a novel *M. tuberculosis* H37Rv three-dimensional microgranuloma model comparing adalimumab and secukinumab side by side. Moreover, *in vivo* mouse *M. tuberculosis* H37Rv infection studies directly comparing anti-IL-17A, anti-IL-17F and anti-TNFα antibody treatments provide further experimental support of the low clinical risk of mycobacterial infection under anti-IL-17A therapy.^37^

Investigating the complex dynamic interplay between the host and the intracellular pathogen *M. tuberculosis* has proven to be challenging. Understanding the host–pathogen interaction during latency and defining the conditions leading to *M. tuberculosis* reactivation has been the subject of numerous studies in various animal species and humans.^48, 49, 50^ The importance of CD4^+^ T cells, TNFα, interferon-γ and IL-12p40, together with the IL-1/IL-1R1 pathway, nitric oxide, reactive oxygen and reactive nitrogen intermediates, in host resistance to intracellular *M. tuberculosis* infection is evident from animal models and human inherited and acquired immunodeficiencies.^50, 51^

The role of TNFα is documented clinically by the association of anti-TNFα therapies with reactivation of LTBIs in psoriasis and rheumatoid indications.^9, 10, 11, 12, 13^ TNFα has an important role in balancing cell survival, apoptosis and programmed necrosis in *M. tuberculosis* infections.^13, 52^ TNFα-mediated apoptosis contributes to host defense by direct antimicrobial effects on intracellular bacilli, and by packaging *M. tuberculosis* and antigens in apoptotic bodies, thereby eliminating the niche for mycobacterial growth.^13, 52^ Hence, anti-TNFα antibody treatment may prevent apoptosis of *M. tuberculosis*-infected macrophages enabling bacterial growth.^13^ Though the importance of TNFα in immunity to *M. tuberculosis* infections has been well studied in various mouse models,^48, 51, 53^ most mouse strains differ from other species (including humans) regarding histopathology and hypoxia of granulomas, a hallmark of TB, and a haven for LTBI.^48, 51, 54^ Thus, to better model the effects of biotherapeutics on aspects of *M. tuberculosis* latency, we utilized a novel *in vitro* human *M. tuberculosis* three-dimensional microgranuloma model to study bacterial dormancy. In view of the complex pathophysiology of *M. tuberculosis* infection, one should be cautious to put clinical latency exactly equivalent to bacterial dormancy.^49^ However, responses to *M. tuberculosis* infection in this *in vitro* model reflect important aspects of host–pathogen dynamics when transitioning into dormancy, characteristics shared with *in vivo* observations in human TB.^38, 39, 40, 41, 42^ These include the following host responses: formation of granuloma structures; occurrence of multinucleated giant cells; lowered CD4 T-cell counts; but increased CD4^+^CD25^+^ T cells; unchanged CD8 T-cell numbers; and increased cytokine and chemokine secretion. When transitioning into dormancy *M. tuberculosis* shows increase of rifampicin resistance, loss of acid-fastness and accumulation of lipid bodies, which can be reverted upon treatment with an anti-TNFα antibody.^38^

This and similar models have been described recently, enabling immunological, biochemical and molecular investigations of host–pathogen interactions during transition from dormant to reactivated pathogen states.^38, 39, 40, 41, 42, 48, 49, 55, 56^ Within few days post infection, the lymphocytes in human *M. tuberculosis*-infected PBMC show clustering around infected macrophages resembling microgranuloma aggregates, a phenomenon not seen in uninfected samples.^38^ *M. tuberculosis* enters into a dormant state in hypoxic granulomas *in vivo*, and dormant mycobacteria are known to exhibit loss of acid-fastness, accumulate lipid bodies and develop rifampicin tolerance as they go into dormancy.^38, 39, 40, 41, 42^ Loss of acid-fastness reflects mycobacterial cell wall reshaping, which facilitates mycobacterial survival in changing host environments, such as hypoxic granulomas in latently infected patients.^57, 58^ A thicker cell wall also restricts the transit of antibiotics, such a rifampicin, across the cell membrane.^46, 57, 58^ The development of drug resistance further strengthens the conclusion that the *in vitro* granuloma model accurately reflects *M. tuberculosis* granulomas in humans. Indeed, in the *in vitro* study, anti-TNFα (adalimumab) treatment showed increased staining for Auramine-O, decreased Nile red staining and decreased rifampicin resistance, indicative of mycobacterial reactivation.^38^ Combined treatment with anti-TNFα antibodies and isoniazid and/or rifampicin may actually provide an opportunity to improve anti-TB therapy.^59^

*M. tuberculosis* infections show increased IL-17A levels in patients with acute TB,^26^ as well as in *in vivo* mouse models^27, 28, 29, 30, 31, 32^ and *in vitro* human PBMC cultures.^33, 34, 35^ Secukinumab treatment in the *in vitro* microgranuloma study—even at 100-fold higher concentrations compared to adalimumab—overall did not show a change of Auramine-O and Nile red staining, and of rifampicin resistance. Secukinumab was comparable to control, which indicated a lack of effect on *M. tuberculosis* dormancy. Although the *in vitro* granuloma model may not be able to capture the entire complexity of IL-17 biology, the lack of effect on *M. tuberculosis* dormancy reported here is in line with the absence of mycobacterial reactivation in clinical and preclinical settings. Further mechanistic investigations into the host–pathogen interactions during transition from dormant to reactivated pathogen states utilizing the *in vitro* human *M. tuberculosis* microgranuloma model are ongoing to address the obvious limitations of this observational study. Whereas anti-TNFα antibody treatment may prevent apoptosis of *M. tuberculosis*-infected macrophages enabling bacterial growth,^13^ a recent study showed that IL-17A actually promotes intracellular growth of *M. tuberculosis* by inhibiting apoptosis of infected macrophages.^60^ Hence, anti-IL-17A treatment may limit intracellular growth of *M. tuberculosis* by enhancing apoptosis of infected macrophages.

Protective and pathological roles of various cytokines and chemokines have been shown in *M. tuberculosis* infections.^19, 20^ A balance of pro- and anti-inflammatory cytokines is thought to be important for bacterial containment in granulomas.^61^ TNFα, interferon-γ, IL-12p40 and IL-1α/IL-1β are of particular importance,^50, 51, 61^ however, the role of IL-17A in different stages of host resistance to *M. tuberculosis* is more equivocal.^18, 19, 20, 21, 22^ The IL-17 pathway seems to be dispensable for low-dose *M. tuberculosis* host resistance in mice infected with the commonly investigated *M. tuberculosis* strain H37Rv,^18, 32^ but, protective immunity to hypervirulent *M. tuberculosis* strain HN878 appears to be IL-17A-dependent in mice.^32^ However, other predominantly intracellular cytokines such as IL-32γ, which occur in humans but not in rodents,^62^ may be more important even against the hypervirulent *M. tuberculosis* strain HN878.^63^ While IL-17A is required for mucocutaneous control of *C. albicans*,^6, 7, 8^ only a small percentage (<5%) of psoriasis patients developed treatable, mild to moderate mucosal and cutaneous candidiasis after a 52-week treatment with secukinumab.^17^ This suggests that infection susceptibility to extracellular fungi such as *C. albicans* is likely a result of a combination of factors rather than a deficiency of just one cytokine, a situation likely to apply as well for host resistance to intracellular pathogens such as *M. tuberculosis.*^19, 20, 21, 22, 61, 64^

In summary, the absence of TB reactivation in secukinumab clinical studies^17^ (and further detailed here) is moreover supported by experimental *in vitro* studies showing lack of effect of secukinumab on *M. tuberculosis* dormancy in a human *in vitro* microgranuloma model (this study), and lack of compromised host resistance in anti-IL-17A-treated *M. tuberculosis*-infected mice.^37^ Collecting real-world evidence data, through registries, will be an opportunity to further substantiate the safety of secukinumab in this regard. Importantly, to date, the composite of clinical, animal and *in vitro* data indicate a low risk for mycobacterial infection under secukinumab therapy, in contrast to anti-TNFα treatment.

## Methods

### Study design and patients

Data were pooled from five randomized, double-blind, placebo-controlled, phase 3 secukinumab studies in 2044 subjects with moderate to severe plaque psoriasis with an overall exposure of 2724.6 patient-years as described separately (ERASURE, NCT01365455; FIXTURE, NCT01358578; FEATURE, NCT01555125; JUNCTURE, NCT01636687; and SCULPTURE, NCT01406938; Figure 1).^17^ The data shown here represent a subset of which baseline demographic and clinical characteristics have been reported in a larger secukinumab long-term safety pooled analysis of 10 phase 2 and 3 clinical studies in patients with moderate to severe plaque psoriasis.^17^ One of these studies (FIXTURE, NCT01358578) included etanercept as the active comparator. A baseline Psoriasis Area and Severity Index score ⩾12 (that is, moderate to severe disease) out of a possible range of 0 (no symptoms) to 72 (worst symptoms) was required for enrollment in each study. Briefly, subjects received secukinumab 300 or 150 mg via subcutaneous injection of reconstituted lyophilisate at baseline, weeks 1, 2, 3 and then every 4 weeks starting from week 4 until week 48. Etanercept 50 mg was given twice weekly to week 12, and then weekly to week 51. In the SCULPTURE study, three secukinumab treatment regimens were compared (each at 300 and 150 mg) to week 52.

### Screening for LTBI before randomization

Subjects with a medical history of active TB, LTBI or positive TB skin test, or LTBI at screening, and receiving secukinumab during 1 year were re-analyzed. Safety data on LTBI or active TB as an adverse event were collected for these subjects across five studies and pooled. Treatment for LTBI was also recorded.

Quantiferon-TB Gold in tube test (QFN-IT; QIAGEN Inc, Germantown, MD, USA), an interferon-γ release assay with high sensitivity and specificity^65^ was used to screen subjects for LTBI before enrollment and randomization. Scheduled testing was performed at the screening visit in all phase 3 trials as outlined in Figure 2. Further Quantiferon tests were permitted at the investigator’s discretion throughout the study. Study inclusion criteria allowed patients positive for LTBI to be randomized to the trial, provided that anti-TB treatment, implemented according to local clinical practice,^43^ the CDC Treatment for Latent Tuberculosis Infection (http://www.cdc.gov/tb/topic/treatment/ltbi.htm) was ongoing or had been completed before randomization. In all, 105 subjects received concomitant anti-TB therapy and secukinumab following LTBI diagnosis at screening or post randomization. Importantly, no subjects discontinued secukinumab treatment while receiving chemoprophylaxis.

### *In vitro* human *M. tuberculosis* H37Rv three-dimensional microgranuloma model

To investigate the effect of adalimumab (anti-TNFα antibody) and secukinumab on *M. tuberculosis* dormancy and reactivation, we utilized a novel *in vitro* human microgranuloma model.^38^ Human PBMCs were isolated from human blood from four healthy volunteers, collected at a blood donation center of the Florida Blood Center in Orlando, FL, USA, as per written informed consent. Florida Blood Centers operate under license from the Food and Drug Administration of the US Department of Health and Human Services. Blood collection and processing was done as per the approval from institutional review board. All donors were tested negative for standard panel of blood-borne pathogens tested by the blood center. PBMCs were purified by Ficoll density gradient separation using standard protocol used for separating PBMCs. After washing, PBMC aliquots of 2 × 10^7^ cells per vial were cryopreserved in 10% dimethylsulfoxide-containing media for extended storage in liquid nitrogen. When needed, PBMC vials were thawed and then washed in RPMI containing 5% human serum. Cells were suspended in RPMI containing 20% human serum and counted by trypan blue dye exclusion method.

PBMC infection by *M. tuberculosis* and granuloma formation was performed as described before.^38^ *M. tuberculosis* H37Rv was cultured in Middlebrook 7H9 medium (supplemented with 10% oleic albumin dextrose catalase (OADC), 0.2% glycerol and 0.05% Tween 80; Difco, Detroit, MI, USA). Cultures containing 2.8 × 10^8^ c.f.u. per ml *M. tuberculosis* was used for all experiments. *M. tuberculosis* cells were suspended in 1 ml of RPMI containing 20% human serum, water bath-sonicated for two pulses of 30 s each and used for infection. An extracellular matrix (ECM) was prepared by mixing 0.95 ml Purecol collagen solution, 50 ml 10 × Dulbecco’s phosphate-buffered saline (Lonza, Allendale, NJ, USA), 4 μl fibronectin (BD Biosciences, San Jose, CA, USA) and 10 μl 1 n NaOH (Sigma, St Louis, MO, USA) per ml of matrix solution and kept on ice (pH 7.0). PBMCs were mixed at room temperature with ECM at 5 × 10^5^cells per 50 μl per well of 96-well plate. Assuming 5% macrophages in PBMCs, *M. tuberculosis* was added to the ECM for infected samples at multiplicity of infection of 1:0.1. ECM was allowed to set by incubating on 37 °C, in CO_2_ incubator for 45 min. Samples were added with RPMI containing 20% human serum and incubated in 37 °C, in CO_2_ incubator. Media were changed on day 7.

After the formation of microgranuloma, anti-TNFα (adalimumab, 40 mg per 0.8 ml; AbbVie AG, Baar, Switzerland), anti-IL-17A (secukinumab, 150 mg ml^−1^ prefilled syringe; Novartis Pharma AG, Basel, Switzerland) antibody or control IgG was added to the media at a concentration of 10 ng ml^−1^. The anti-IL-17A (secukinumab) antibody was also tested at 100 and 1000 ng ml^−1^. Antibody treatments were performed in an anonymized manner. As indicator of *M. tuberculosis* dormancy we determined loss of acid-fastness and accumulation of lipid bodies on day 8, by staining with Auramine-O and Nile Red staining I (TB Fluorescent Stain Kit M, BD Biosciences, San Jose, CA, USA), as described.^38^ Slides were mounted in cytoseal with coverslip and viewed under the Nikon TE2000 fluorescent microscope (Nikon Corp., Tokyo, Japan). Images were acquired using a cool snap HQ^2^ camera (Photometric, Tuscon, AZ, USA) or a Nikon Digital Sight DS Ri1 camera. ‘NIS elements’ software (Nikon) was used for acquisition of images. Images were taken using the Texas red filter set and the FITC filter set (Chroma, Rockingham, VT, USA).

As dormant mycobacteria show increased rifampicin resistance,^38, 39, 40, 46^ granuloma samples were either treated with rifampicin (dissolved in dimethylsulfoxide) or left untreated (control) (Sigma; rifampicin, 5 μg ml^−1^ final concentration).^38, 39^ On day 8 or 9 post infection, media was removed, replaced with 100 μl per well of media containing rifampicin and incubated for 3 days. Then, media was removed and wells treated with 50 μl per well collagenase (Sigma) for 40 min at 37 °C to isolate host PBMC cells. Samples from five wells were pooled in 1.8 ml micro centrifuge tubes and host cells were lysed with 200 μl of 0.1% Triton X-100 solution. *M. tuberculosis* pellet was obtained by centrifuging at 3500 *g* for 12 min. The *M. tuberculosis* pellet was suspended in 1 ml 7H9 media and 10-fold serial dilutions were made in Middlebrook 7H9 media containing 0.05% Tween 80 and 100 ml samples plated on Middlebrook 7H10 agar plates. Plates were incubated at 37 °C. The c.f.u. were determined after 4 weeks. % Rifampicin tolerance is calculated by formula: %Rif-tolerance=c.f.u.(Rif)/c.f.u.(untreated) × 100.

### Statistical analysis

Data are represented as median with interquartile ranges, and statistically evaluated by multiple comparisons with a two-way analysis of variance (GraphPad Software Prism 7, La Jolla, CA, USA). *P*-values <0.05 were considered significant.

## Figures and Tables

**Table 1:** Subjects with a medical history of tuberculosis or LTBI showed no reactivation of tuberculosis during secukinumab treatment across five clinical studies

*Treatment*	*Subjects*		*Anti-tuberculosis medication*	*Secukinumab (mg)*^a^	*Median duration of secukinumab treatment (days)*	*Active tuberculosis*
Quantiferon tested positive	107	52	Yes	150	364	0
		55	Yes	300	364	0
Quantiferon tested negative	25	14	No	150	362	0
		11	No	300	363	0

Abbreviation: LTBI, latent tuberculosis infection.

a Subjects received secukinumab 300 or 150 mg subcutaneously at weeks 0, 1, 2 and 3, and then every 4 weeks starting from week 4.

**Table 2:** Subjects in phase 3 secukinumab trials diagnosed with LTBI in screening and on anti-tuberculosis therapy concurrently show no serious adverse liver events

*Newly occurring or worsening liver enzyme abnormality*	*Any secukinumab dose (*n=*105),*n*/*m*(%)*
ALT >5 × ULN	3/104 (2.9)
ALT >8 × ULN	1/104 (1.0)
ALT >10 × ULN	0/104 (0.0)
ALT >20 × ULN	0/104 (0.0)
AST >5 × ULN	2/103 (1.9)
AST >8 × ULN	0/103 (0.0)
AST >10 × ULN	0/103 (0.0)
AST >20 × ULN	0/103 (0.0)
TBL >1.5 × ULN	2/103 (1.9)
TBL >2 × ULN	1/104 (1.0)
TBL >3 × ULN	0/104 (0.0)

Abbreviations: ALT, alanine transaminase; AST, aspartate transaminase; LTBI, latent tuberculosis infection; *m*, number of subjects at risk for an abnormality with non-missing value at baseline and post baseline; *n*, number of subjects who meet the designated criteria; TBL, total bilirubin; ULN, upper limit of normal.
